# Extension of the sasCIF format and its applications for data processing and deposition

**DOI:** 10.1107/S1600576715024942

**Published:** 2016-02-01

**Authors:** Michael Kachala, John Westbrook, Dmitri Svergun

**Affiliations:** a Hamburg Outstation, European Molecular Biology Laboratory, Notkestrasse 85, Hamburg 22607, Germany; b RCSB PDB, Department of Chemistry and Chemical Biology and Center for Integrative Proteomics Research, Rutgers, The State University of New Jersey, Piscataway, NJ 08854, USA

**Keywords:** sasCIF, CIF, small-angle scattering, computer programs

## Abstract

Recent advances in small-angle scattering (SAS) experimental facilities and data analysis methods have prompted a dramatic increase in the number of users and of projects conducted, causing an upsurge in the number of objects studied, experimental data available and structural models generated. To organize the data and models and make them accessible to the community, the Task Forces on SAS and hybrid methods for the International Union of Crystallography and the Worldwide Protein Data Bank envisage developing a federated approach to SAS data and model archiving. Within the framework of this approach, the existing databases may exchange information and provide independent but synchronized entries to users. At present, ways of exchanging information between the various SAS databases are not established, leading to possible duplication and incompatibility of entries, and limiting the opportunities for data-driven research for SAS users. In this work, a solution is developed to resolve these issues and provide a universal exchange format for the community, based on the use of the widely adopted crystallographic information framework (CIF). The previous version of the sasCIF format, implemented as an extension of the core CIF dictionary, has been available since 2000 to facilitate SAS data exchange between laboratories. The sasCIF format has now been extended to describe comprehensively the necessary experimental information, results and models, including relevant metadata for SAS data analysis and for deposition into a database. Processing tools for these files (*sasCIFtools*) have been developed, and these are available both as standalone open-source programs and integrated into the SAS Biological Data Bank, allowing the export and import of data entries as sasCIF files. Software modules to save the relevant information directly from beamline data-processing pipelines in sasCIF format are also developed. This update of sasCIF and the relevant tools are an important step in the standardization of the way SAS data are presented and exchanged, to make the results easily accessible to users and to promote further the application of SAS in the structural biology community.

## Abstract 2

An extension of the sasCIF format is intended for representation, storage and exchange of SAS data and SAS-based models. Open-source *sasCIFtools* are developed for convenient data conversion, and for deposition into and retrieval from databases and data-processing pipelines.

## 1. Introduction: motivation for sasCIF update   

The broad availability of advanced experimental facilities for small-angle scattering (SAS) of X-rays (SAXS) and neutrons (SANS), together with novel data analysis methods, have prompted a dramatic increase in the popularity of the method, most notably its use in investigating the overall structures of biological macromolecules in solution (Graewert & Svergun, 2013 ▸). This increase has led to an upsurge in the number of objects studied, the experimental data available and the structural models generated. To accommodate these data and make them more accessible to the community (Trewhella *et al.*, 2013 ▸), biological SAS databases were introduced and are being further developed, for example BIOISIS (http://www.bioisis.net at the Lawrence Berkeley National Laboratory; Hura *et al.*, 2009 ▸) and the Small-Angle Scattering Biological Data Bank or SASBDB (http://www.sasbdb.org, developed at EMBL Hamburg; Valentini *et al.*, 2015 ▸).

Both databases contain SAS data and models, but currently the means of information exchange with and between the databases are not established and this may cause problems in data management, leading to duplications and incompatible frameworks. The lack of agreed standards with respect to data deposition also limits and complicates the development of data mining and analysis protocols, thus creating obstacles for future data-driven research. Finally, cross-platform exchange of experimental data, analysis protocols, general experimental information, and the results obtained using various instruments and radiation sources (X-rays or neutrons) is hindered by the lack of a consistent and user-friendly file structure.

Discussions on this issue within the SAS community resulted in the recommendation by the Task Forces on SAS and hybrid methods for the International Union of Crystallography and the Worldwide Protein Data Bank (wwPDB) to develop a federated approach to SAS data and model archiving. Within the framework of a federated approach, the existing databases may exchange information and provide independent but synchronized entries to users using a standard file format for SAS data exchange that includes all relevant information stored in the databases (Trewhella *et al.*, 2013 ▸).

In this publication, we present a solution developed to avoid inconsistencies and to provide a universal SAS data exchange protocol for the community. This protocol is based on the widely adopted crystallographic information framework (CIF) and was first introduced for SAS in 2000 (Malfois & Svergun, 2000 ▸) to facilitate the exchange of one-dimensional SAS data between laboratories. However, SAS databases store other types of information in addition to the scattering data, including a number of overall structural parameters, data transforms [*e.g.* Guinier (1939 ▸) and Kratky (Glatter & Kratky, 1982 ▸) plots], real-space pair-distance distributions, various types of structural model for the particle(s) in question and the relevant fits of the computed scattering fits to the experimental data. We have extended the original specification of sasCIF to describe comprehensively the required types of data plus the metadata information needed for SAS projects. The extended format is useful for database deposition, and also for data storage and exchange between facilities and scientists. In order to make the format accessible to members of the community, convenient tools have been developed to handle and process the data (*sasCIFtools*). The tools include sasCIF converters for the results generated by popular analysis programs (both current and some older versions), making the data and metadata formatting and database deposition a seamless process. *sasCIFtools* are available as standalone programs and are integrated into the SASBDB database, allowing export and import of the data entries as sasCIF files. Further, the tools are integrated into the *SASFLOW* SAXS data-processing pipeline (Franke *et al.*, 2012 ▸), allowing online storage of the experimental data as sasCIF files.

## 2. General idea and basic concepts of sasCIF   

sasCIF is built on CIF (Hall *et al.*, 1991 ▸), a system of standards and specifications for the exchange and archiving of structural data and models. CIF is the *de facto* standard data exchange protocol for small-molecule X-ray diffraction experiments (Hall *et al.*, 1991 ▸). The macromolecular crystallographic information framework (mmCIF) (Westbrook, Yang *et al.*, 2005 ▸) is similarly used to describe macromolecular structural experiments, and the PDBx/mmCIF (Westbrook, Henrick *et al.*, 2005 ▸) extension is used by the wwPDB for archiving and data management.

The CIF data architecture consists of three layers. The properties of data items used in data files are defined in a domain data dictionary. The metadata properties used in the domain dictionary are in turn defined in a core metadata dictionary describing the dictionary definition language (DDL) (Westbrook & Hall, 2005 ▸; Westbrook *et al.*, 2005 ▸). Data files and dictionaries are all expressed in a common syntax based on the self-defining text archival and retrieval (STAR) format (Hall, 1991 ▸; Hall & Spadaccini, 1994 ▸).

The sasCIF domain dictionary presented in this publication follows this CIF protocol. The content and organization of the sasCIF dictionary have been developed to be compatible with PDBx/mmCIF. A detailed description of the structure elements used in sasCIF files is presented in the following sections.

A sasCIF data file follows the mmCIF syntax, consisting of a small number of syntax elements. The simplest element consists of a paired collection of data item names and values. mmCIF data item names are identified by a leading underscore character. The underscore is followed by a text string, interpreted in mmCIF as containing both a category name and a keyword name separated by a period. The keyword portion of the name is the unique identifier of the data item within the category. For example, in the data item name _sas_beam.id, sas_beam is a category name and id is the keyword name.

Short text strings may be enclosed in single or double quotation marks. Text strings which span multiple lines are enclosed by semicolons, which are placed at the first character position of the line. There are two special characters used as placeholders for any mmCIF item values which, for whatever reason, cannot be explicitly assigned. The question mark (?) is used to mark an item value as missing, and a period (.) may be used to identify that there is no appropriate value for the item or that a value has been intentionally omitted.

Vectors and tables of data may be encoded in mmCIF using a loop_ syntax directive. To build a table, the data item names corresponding to the table columns are preceded by the loop_ directive and followed by the corresponding rows of data. The use of the loop_ directive in mmCIF has a few restrictions. First, it is required that all of the data items within the loop belong to the same mmCIF category. Second, the number of data values following the loop must be an exact multiple of the number of data item names.

mmCIF uses data blocks to organize related information and data. A data block is a logical partition of a data file created using a data_ directive. A data block may be named by appending a text string after the data_ directive and a data block is terminated either by another data_ directive or by the end of the file.

## 3. Structure and content of the extension   

Various types of data and metadata are required to describe SAS experiments and data analysis processes. The two-dimensional scattering patterns registered by a detector are reduced to one-dimensional scattering profiles, from which the overall parameters and real-space pair-distance distribution functions are calculated, and various *ab initio* and hybrid models and their fits against the experimental data are generated. General metadata about the sample and buffer and about the experimental setup and conditions are important as well. All of these kinds of data, except for the two-dimensional images (which can be represented with the imgCIF format; Bernstein & Hammersley, 2005 ▸), are included in the SAS databases, but not all data types were defined in the previous sasCIF dictionary (Version 0.4; Malfois & Svergun, 2000 ▸). That version included one-dimensional scattering data as well as information about the beam, detector and sample. To determine which data items and categories to incorporate into the extended sasCIF dictionary, the contents of the SASBDB and BIOISIS databases were examined. It was found that the following information should be included in the sasCIF 0.5 dictionary to address the needs of the present, and potentially also the future, SAS user community:

(i) results of SAS measurements, including concentration and/or contrast variation series information;

(ii) results obtained for the normalization of scattering intensities, including absolute scaling or scaling relative to secondary standards;

(iii) Guinier plot analysis (Guinier, 1939 ▸) and the calculated real-space distance distribution functions (Glatter, 1977 ▸);

(iv) overall parameters derived from the experimental data; radius of gyration (*R*_g_), extrapolated scattering intensity at zero angle [*I*(0)], maximum dimension of the particle (*D*_max_) and, importantly, molecular mass (*M*_r_) information extracted from various approaches [from normalized *I*(0), Porod volume, *ab initio* volumes *etc.* (Mylonas & Svergun, 2007 ▸; Svergun *et al.*, 2013 ▸)];

(v) a description of the macromolecular samples, including sequence information;

(vi) information about the sample environment, including supporting solvent composition, temperature, pH, contrast *etc.*;

(vii) spatial (three-dimensional) models and calculated model scattering fits to the data, with statistical reporting of data–model discrepancies;

(viii) author names, affiliations and publication information;

(ix) cross-database links and information, including Uniprot (for proteins), PubMed and the Protein Data Bank (PDB).

**Figure 1:**
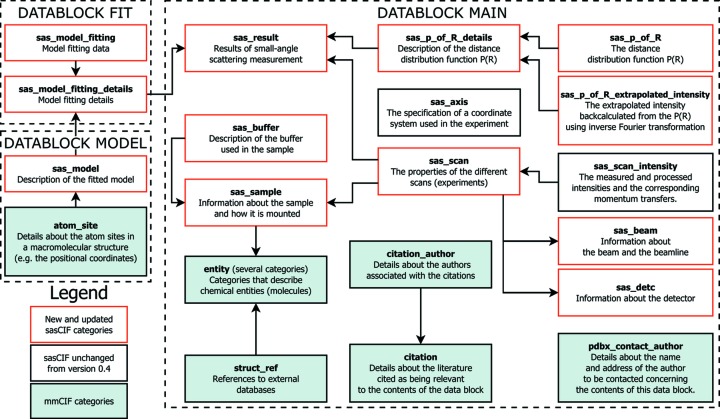
Relations between categories in the updated sasCIF dictionary. The categories that remained unchanged from the previous version(s) are shown in black boxes, while updated ones are shown in red boxes. Categories from the mmCIF dictionary are in boxes with a pale-green background.

A relational diagram of the updated version of sasCIF is presented in Fig. 1 ▸ (with categories and their description) and supplementary Fig. S1 (with all data items); the category groups are shown in supplementary Table S1. In order to accommodate all the required information, new categories and data items have been added to the sasCIF dictionary, including categories adopted from the mmCIF dictionary. Some existing sasCIF categories have been supplemented with new data items, while others were completely redesigned as new categories. In parallel with these changes, updates of parent–child relationships between the categories have been implemented (supplementary Table S2). The updated sasCIF dictionary (version 0.51) is available at http://mmcif.wwpdb.org/dictionaries/mmcif_sas.dic/Categories/index.html.

### 3.1. New categories incorporated into sasCIF   

sas_result: this category contains information about the overall parameters and their experimental errors, *e.g.* radius of gyration, molecular mass, maximal dimension *etc.*

sas_p_of_R, sas_p_of_R_extrapolated_intensity, sas_p_of_R_details: a set of categories that describe the pair-distance distribution [*p*(*r*) *versus r*]. The three-element structure of the category is based on the typical structure of files containing the pair-distance distribution (*e.g.* *GNOM*; Svergun, 1992 ▸), which includes the real-space distance distribution (sas_p_of_R) and the reciprocal-space fit of the extrapolated intensities (calculated *via* Fourier transform) to the experimental scattering curve (sas_p_of_R_extrapolated_intensity). The distance distribution information is divided into three categories, because according to CIF syntax the loop_ structures, such as real-space *p*(*r*) and reciprocal-space extrapolated intensities, and the supporting metadata (sas_p_of_R_details) must be stored in separate categories.

sas_model_fitting, sas_model_fitting_details: categories that store the fitted calculated model curve (sas_model_fitting), as well as a description of the details of the calculated model against the data and the statistical assessment of the fit, such as its χ^2^ value (sas_model_fitting_details).

sas_model: the category describing the properties of the refined model used to interpret the SAS data. The category includes the model type [*ab initio* (Svergun, 1999 ▸) or hybrid rigid body (Petoukhov & Svergun, 2005 ▸)], the software used to build/refine the model, the model symmetry (*P*1, *P*2 *etc.*) and, in the case of *ab initio* models, the radius of the individual bead atoms used to represent the shape of the particle.

sas_buffer: the buffer/solvent description, including small-molecule components, concentration, pH *etc.*

### 3.2. Existing sasCIF categories that have been expanded   

sas_sample: new data items added to describe the sample component macromolecule are the UV-absorption extinction coefficient, partial specific volume, dry volume, molecular mass, additional methods used to assess data quality (*e.g.* size exclusion profiles, gel electrophoresis images), sample name and sample concentration. Given that sasCIF should be valid for both SAXS and SANS experiments, X-ray and neutron contrast and the level of isotopic labelling (perdeuteration) of the macromolecule are also included. Finally, the sas_sample category contains a pointer to entity categories (as defined in mmCIF) describing the properties of the molecules which constitute the sample.

sas_detc: data items for the name and type of detector have been added, for example a photon-counting or CCD detector.

sas_scan: the experimental parameters were extended to include temperature data (during sample storage and data collection), the number of data frames taken to comprise the final data set, the units of momentum transfer (inverse ångströms or inverse nanometres) and the experimental momentum transfer range.

sas_beam: the new data items contain information about the beamline, its name, its geographical location and the type of source.

### 3.3. Categories from mmCIF used in sasCIF files   

atom_site: atomic coordinates of spatial models. Atomic or *ab initio* models included in sasCIF files can be displayed with molecular visualization software such as *RasMol*/*RasWin* (Sayle & Milner-White, 1995 ▸), *Jmol* (Hanson, 2010 ▸) and *PyMOL* (Schrödinger, 2010 ▸).

entity, entity_name_common, entity_poly, entity_src_gen: categories used to describe molecules in the sample.

struct_ref: references to external databases, in this case UniProt (for proteins).

citation, citation_author: publication information, including cross-links to PubMed.

### 3.4. Structure of a sasCIF file   

The data blocks in a sasCIF file are not described in the dictionary, but instead they are defined by the dictionary structure. A category can be present in a data block only once, so in cases where several models are presented that fit one and the same SAS profile, each model must be stored in a separate data block. As seen from supplementary Table S2, there are four categories with one-to-many relationships in the dictionary. Two of them, entity and citation_author, can be described with the loop structure, while the other two (sas_model_fitting_details and sas_model) require dedicated data blocks (FIT and MODEL, correspondingly) contained in the same file for each model or fit. Every other category is unique within the framework of a sasCIF file and is stored in the MAIN data block. The final data block structure of the new sasCIF format is shown in supplementary Fig. S2. If there is a need to store several scattering curves in a single sasCIF file (*e.g.* for concentration series or contrast variation), the file may also contain several MAIN data blocks.

## 4. Tools for creation and processing of sasCIF files   

This update of the sasCIF dictionary is an important step towards the efficient organization and accessibility of SAS data and associated experimental information encompassing the aspects of data analysis and sample quality assurance. To make use of the sasCIF files, user-friendly and convenient tools for sasCIF processing and integration into existing software and services are crucial. In this section we describe *sasCIFtools*, a set of python scripts for data processing and format conversion, as well as the implementation of sasCIF support for SASBDB and for the SASFLOW automated data-processing pipeline (Franke *et al.*, 2012 ▸).

### 4.1. Description of *sasCIFtools* and the data types that can be processed   

The purpose of *sasCIFtools* is to provide the means to convert data into sasCIF files *via* the addition and extraction of SAS information from commonly used data formats. As discussed above, sasCIF files include several types of data:

(i) metadata about the sample, experimental conditions, results of the experiment *etc.*;

(ii) experimental scattering data;

(iii) real-space pair-distance distribution [*p*(*r*)];

(iv) spatial (three-dimensional) models;

(v) fit(s) of calculated scattering intensities from spatial models to the experimental data.

The programs used in SAS data analysis have different input requirements and output file structures, depending on the operations required for the particular analysis: probable pair-distance distributions (*e.g.* generated by *GNOM*; Svergun, 1992 ▸), dummy-atom bead models and fits (*e.g.* built by *DAMMIN*; Svergun, 1999 ▸), atomic model fits to experimental data (*e.g.* using *CRYSOL*; Svergun *et al.*, 1995 ▸) *etc*. Except for metadata, the types of input data and the output file structure are pre-defined by each individual software module. The aim of *sasCIFtools* is to read these software-dependent outputs and collate them into a sasCIF file with the relevant information for the entire experiment or for a collection of several experiments (*e.g.* for a concentration or pH series) and with the corresponding data analysis results. The *sasCIFtools* support all major input and output formats of one of the most widely used SAS data analysis and modelling suites, *ATSAS* (Petoukhov *et al.*, 2012 ▸).

#### 4.1.1. Tools for the scattering data processing   

The experimental data are typically stored in a three-column ASCII format (usually with the extension .dat) containing the momentum transfer *s*, scattering intensities *I*(*s*) and experimental errors σ*I*(*s*). In some cases, the files also contain the metadata of the experiment (*e.g.* temperature, number of frames, sample concentration) in the form of name–value pairs. To transfer the scattering data to a sasCIF file, the experimental stream should be stored in the sas_scan_intensity category and the relevant metadata in sas_scan, sas_sample and other categories. The detailed relationship between the .dat file content and the sasCIF categories is shown in supplementary Table S3.

The experimental scattering profile is the most basic and essential piece of information for SAS data analysis. The *dat2cif* tool has been designed to add experimental data in .dat format to sasCIF files. The *cif2dat* tool extracts *s*, *I*(*s*) and σ*I*(s) from the sasCIF file, along with experimental parameters (momentum transfer *s* and its units) and experimental information (sample storage and data collection temperature and, if applicable, the number of data frames used for averaging).

#### 4.1.2. Tools for processing real-space pair-distance distributions [*p*(*r*) *versus* *r*]   

One of the major steps in SAS data analysis is the calculation of the real-space distance distribution function which, in the *ATSAS* package, is done by the program *GNOM* (Svergun, 1992 ▸). The *GNOM* output (the .out file) contains the real-space distance distribution *p*(*r*) for *r* values between 0 and *D*_max_, the regularized and smoothed reciprocal-space fit of *p*(*r*) to the scattering data, the fit extrapolated to *s* = 0, and the structural parameters extracted from the distribution [*R*_g_ and *I*(0)]. The distance distribution is stored as a three-column table [the intraparticle distance *r*, the value of *p*(*r*) and the estimated error of this value σ*p*(*r*)]. The associated fit of *p*(*r*) in reciprocal space against the scattering data is displayed in a five-column format [scattering vector *s*, experimental intensity *I*(*s*), experimental error σ*I*(*s*), the regularized fit of the intensities against the experimental data *I*_reg_(*s*), and the scattering intensity desmeared and extrapolated to *s* = 0, *I*_ext_(*s*)]. In sasCIF, the content of the .out file is stored in three separate categories: sas_p_of_R is used for the distance distribution, sas_p_of_R_extrapolated_intensity for the extrapolated scattering curve and sas_p_of_R_details for other information extracted from the .out files. The .out file with the pair-distance distribution is read by *out2cif*, which adds the distance distribution [*r*, *p*(*r*), σ*p*(*r*)] and the associated information (momentum transfer *s* and extrapolated regularized fit), as well as the relevant parameters that, when combined, are required to reconstruct the distribution from the resulting sasCIF file.

The inverse procedure, extraction of a .out file from sasCIF to reconstruct the pair-distance distribution and the regularized reciprocal-space fit of *p*(*r*) *versus r* to the data, is not trivial. For some .out files, information regarding how the extrapolated fit to the data was obtained to perform the indirect Fourier transform to obtain *p*(*r*) *versus r* is not available. The reason for this is that automated data manipulations performed by *GNOM* may require one to optimize the calculation performance, *e.g.* in cases where the number of points in the input data is too large, *GNOM* will re-bin the data with a larger angular step, thus reducing the number of data points for the calculation. As a result, the Δ*s* increment of the experimental data and that used in the .out file may differ from each other. To resolve this issue in the *cif2out* tool, the intensities of the .out file are translated back to the same Δ*s* (same bin sizes) as the initial scattering curve and then added to the sasCIF-extracted .out file.

#### 4.1.3. Tools for processing spatial (three-dimensional) models   

Spatial models (*ab initio*, rigid body or ensembles of models) refined against or built based on SAS data are often stored in a Brookhaven Protein Data Bank (PDB) format as .pdb files. The most reasonable approach to handling three-dimensional spatial coordinates is to employ dedicated mmCIF structures to store the information in sasCIF. The category in mmCIF that contains atomic coordinates is atom_site and the correspondence between .pdb fields and mmCIF data items is straightforward, as defined at the PDBx/mmCIF Dictionary Resources web site (http://mmcif.wwpdb.org/docs/pdb_to_pdbx_correspondences.html). For inserting the atomic coordinates obtained from spatial models, *e.g.* from a .pdb file, the module *pdb2cif* was developed. The tool reads the input .pdb file in text format and transfers coordinates and other structural information from it to the atom_site category of the output sasCIF file. Spatial models consisting of atomic coordinates are extracted from the sasCIF files with the *cif2pdb* sasCIFtool, which writes data from the atom_site category into a standard .pdb file. As the sasCIF file may contain several models, each in its own data block, the individual models are saved into separate files.

#### 4.1.4. Tools for processing the fits by spatial models against SAS data   

The scattering calculated from three-dimensional models is routinely used during model refinement and the refined data–model discrepancies are reported, usually as a reduced χ^2^ value, or more recently using a *p* value, which is a goodness-of-fit estimator obtained from the correlation map test applied to one-dimensional scattering curves (Franke *et al.*, 2015 ▸). Files containing the model fit information are four-column .fir files for *ab initio* models and three-column .fit files for high-resolution model fitting. Both file formats store momentum transfer *s*, intensity *I*(*s*) and calculated model scattering *I*_fit_(*s*) [in addition, the .fir files also contain experimental errors Err(*s*)]. Unlike .out files, the .fit or .fir files do not store scaling or cropping information, and so the original experimental scattering intensities used for the refinement can in general not be reconstructed from the .fit files alone. In sasCIF, the fits of spatial models against the data are represented by two categories: sas_model_fitting containing *s*, *I*(*s*) and *I*_fit_(*s*), and sas_model_fitting_details with information on experimental units, reduced χ^2^ and/or *p* values. The experimental errors are stored together with the original scattering data in the sas_scan_intensity category. The insert tool *fit2cif* handles the insertion of data from .fit or .fir files into sasCIF files. The calculated scattering from a model and its fit against the data are extracted by *cif2fit*, which writes the scattering profiles and reporting statistics (reduced χ^2^ and *p* value) to the output .fit file.

#### 4.1.5. Tools for the processing of meta-information and all available data   

In addition to the information that can be stored in the data files, sasCIF contains information about the properties of the sample, the solvent, parameters of the experiment, publication records and other meta-information. To extract this information, a *cif2sub* tool was designed that writes all metadata to a text file as name–value pairs. The metadata values written to this file are chosen to match those requested in the submission process to SASBDB.

Finally, the tool *cif2all* extracts all available data from a sasCIF file at once. *cif2all* calls the corresponding functions from the other sasCIF extraction tools and saves the individual output files in either the current folder or a user-specified directory.

#### 4.1.6. External and internal libraries used by *sasCIFtools*   

For *sasCIFtools* to perform their tasks, *i.e.* to convert SAS data files and .pdb files to sasCIF and *vice versa*, external libraries for reading and writing the relevant files have been used and additional libraries to cover non-standard operations have been developed. *PDBeCIF* provided by EMBL-EBI (https://github.com/glenveegee/PDBeCIF) is utilized to read/write sasCIF files, while for *ATSAS* file processing the *saxsdocument* library is used, which was initially written as part of the *saxsview* project at EMBL-Hamburg (http://saxsview.sourceforge.net/). Both these libraries have been modified to process sasCIF files using the updated data type requisites. In operation, the *sasCIFtools* require an installed *ATSAS* package (Petoukhov *et al.*, 2012 ▸) and Python 2.7 (https://www.python.org/download/releases/2.7/). The individual libraries of the new sasCIF package are described below.

The *PDBeCIF* library is designed to read and write mmCIF files. The library is written in pure Python, has no external dependencies and is distributed under the Apache 2.0 License. Therefore, the package is easy to modify, allowing for flexible application. Although *PDBeCIF* was initially developed to process mmCIF files, it does not impose restrictions on specific categories or data items. Consequently, the library can be used with any format from the CIF family, including sasCIF. The CIF structures are read and stored as nested Python dictionaries, *i.e.* each CIF file is a dictionary of data blocks, each data block is a dictionary of categories and each category is a dictionary of data items with item names as keys. According to the STAR format, the order of structures in a CIF file, *i.e.* the order of categories in a data block, is irrelevant, and the same holds for the order of the items in a generic Python dictionary. However, to make the sasCIF files more readable for the user, the order of elements should be predictable. To achieve this, the *PDBeCIF* package was modified for *sasCIFtools* by using an ordered dictionary structure instead of a generic one. This structure saves the items in the sequence they were assigned, and therefore the order is determined within the individual *sasCIFtools* programs.

*saxsdocument* is a part of the *saxsview* project (http://saxsview.sourceforge.net/) that provides input/output and plotting libraries for SAS data files (.dat, .out, .fir and .fit). The library is available for C, Fortran and Python, but in the last case it is implemented as read-only. To provide an option for writing SAS data files with Python, an internal library *writesaxsdoc* was developed for *sasCIFtools*. It allows the writing of properties for name–value pairs and tabular data, *e.g.* column-format scattering profiles and fits.

*cifutils* is an additional internal *sasCIFtools* library that contains support functions for all *sasCIFtools*, for example, argument handling.

*sasCIFtools*, along with the user manuals, are available as open source Python scripts and modules at https://github.com/emblsaxs/sasciftools.

### 4.2. Integration with SASBDB   

SASBDB is at present a standalone data bank, but it may in future join a federated system of databases for biological SAXS and SANS data (Trewhella *et al.*, 2013 ▸). The information stored in SASBDB contains experimental scattering curves, structural parameters, distance distributions, modelling results and model fits, plus additional information about the authors, sample environment and experimental installation. SASBDB is a relational MySQL database, where each entry (usually corresponding to an experimental scattering curve) is identified by a seven-letter alphanumeric code starting with SAS.

In order to import data from the SASBDB in sasCIF format, we first needed a direct way of extracting information from the database. For this purpose the Python library *MySQLdb* (Dustman, 2013 ▸) was used and a *sql2cif* tool was developed. The library establishes a connection with the MySQL database and processes the SQL queries. *MySQLdb* fetches either one or all entries from the database that satisfy the query. For convenience and for further manipulations, the fetched results are stored in a nested Python dictionary that consists of table dictionaries, each element being a field name–field value pair. The *sql2cif* tool translates the data from that dictionary to a sasCIF-like Python dictionary as specified by the *PDBeCIF* library. Each sasCIF data block (MAIN, FIT and MODEL) and separately the pdbx_contact_author and citation categories are processed independently using dedicated functions in *sql2cif*, providing additional flexibility to use this result with other *sasCIFtools*.

The correspondence between the structure and field names of SASBDB differs from the structure and data item names of sasCIF. As both SASBDB and the extended sasCIF format are still under development, it is helpful to keep this correspondence flexible, and a dedicated dictionary was developed. The dictionary is a plain text file with the correspondence specified for the tables and fields of SASBDB. If there are any changes to either SASBDB or sasCIF, they can easily be included in bdb2cif.dic without modifications of the *sasCIFtools* or SASBDB code.

Although most of the fields from SASBDB can be translated directly to sasCIF data items, some fields still require preprocessing. Among such cases is a description of the oligomeric state of a macromolecule or complex, which, at present, has to be converted from a word (*e.g.* dimer) into a number (in this case, 2). Another example is the primary sequence of the macromolecule, *e.g.* a protein sequence, presented in one-letter FASTA format in SASBDB, while sasCIF employs a plain one-letter code without comments. More complicated preprocessing is performed for the citation information, which is saved as plain text in the database and has to be parsed and stored as several data items in sasCIF (journal name, volume, issue, pages *etc.*)

**Figure 2:**
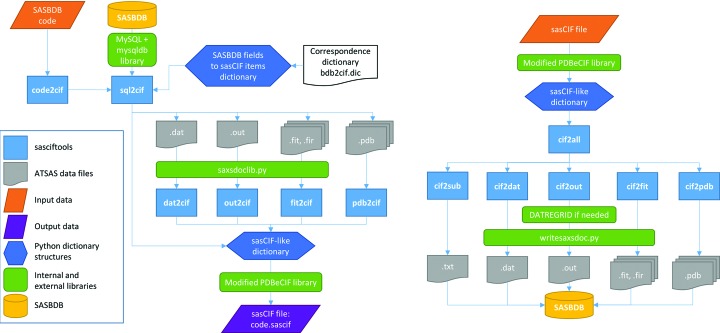
The integration of *sasciftools* with SASBDB. (*a*) Export from the database. (*b*) Import to the database.

*sql2cif* as such does not have a user interface; instead, the user’s interaction with the database is facilitated by a *code2cif* tool, which manages data export from SASBDB to a sasCIF file. The operation of *code2cif* illustrated schematically in Fig. 2 ▸(*a*) starts with the user providing an input SASBDB code. Then, *code2cif* calls *sql2cif* and retrieves all the information relevant to the entry with this code. Part of the information is added directly to the sasCIF-like Python dictionary and the location of the data files is communicated to other *sasCIFtools* called by *code2cif*. These tools add the scattering curve, distance distribution, models and model fits to the same dictionary. Finally, this dictionary is written to the output sasCIF file using the modified *PDBeCIF* library. The inverse process of importing data to the database is shown in Fig. 2 ▸(*b*). The import procedure is fully managed by the *cif2all* tool, which reads the input sasCIF file into the Python dictionary and then calls the tools described above. The *sasCIFtools* scripts provide all the necessary files, but do not write any information directly to the database, because that process is handled directly by SASBDB, either manually or automatically.

### 4.3. Integration with *SASFLOW*   

One of the reasons for the rapidly increasing amount of SAS data is the implementation of robotic data-collection procedures followed by automated reduction and processing tools (Graewert & Svergun, 2013 ▸). In particular, an automated system called *SASFLOW* (Franke *et al.*, 2012 ▸) is employed on the EMBL Biological SAXS P12 beamline at the PETRA-3 synchrotron (Hamburg). The pipeline is linked to a robotic sample loader and performs primary data reduction and initial processing and analysis, including the determination of overall parameters, calculation of the distance distribution function and *ab initio* modelling. For each measured sample, *SASFLOW* provides sufficient information to describe the experimental conditions, the scattering data, the overall parameters and the preliminary shape of the solute. At present, the overall parameters are saved in an XML pivot table, and other types of data and models are stored in several separate files. To facilitate archiving and exchange of the data, a software component was developed to generate a single sasCIF file for each sample measurement.

**Figure 3:**
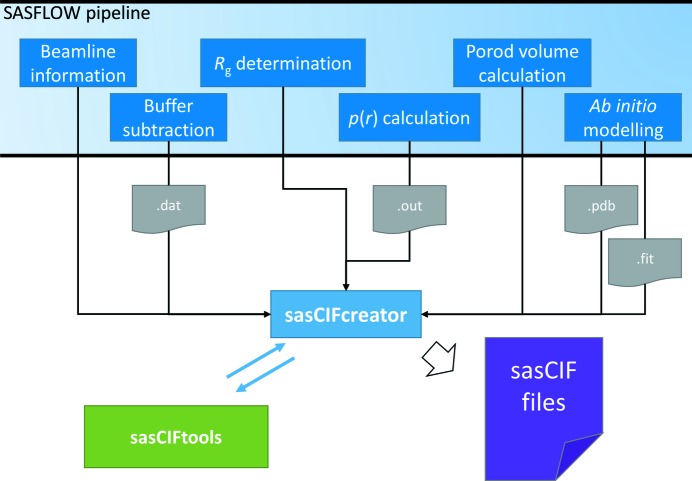
Integration of *sasCIFtools* with the *SASFLOW* pipeline *via* the *sasCIFcreator* component.

The sasCIF files are created by the extra pipeline component *sasCIFcreator*, which collects all data generated during the *SASFLOW* run and puts it onto a single sasCIF file for each measurement. *sasCIFcreator* takes meta-information about the sample and experiment and the location of the data files from the pipeline storage. The metadata are written to a designated sasCIF file by *sasCIFcreator* itself, and the corresponding *sasCIFtools* extract data from the files containing the experimental scattering curve, the distance distribution function, the *ab initio* model and its theoretical scattering fitted to the experimental data. The process of data gathering is shown in Fig. 3 ▸. The sasCIF file generated after each run can be used as a starting point for the preparation of a sasCIF file to be deposited in SASBDB or other databases.

### 4.4. Current and potential future applications of sasCIF   

sasCIF is capable of accommodating all presently required types of SAS information, and in future it could be used as both an archival file format and a SAS project file that accumulates data during the course of a data analysis project (essentially, a sasCIF ‘on-the-fly’ record of data processing and analysis). A possible way of implementing on-the-fly sasCIF is presented in supplementary Fig. S3. In the first step, the scattering data and relevant experimental and selected structural parameters (*R*_g_, molecular mass *etc.*) are added to a sasCIF file automatically, *e.g.* using a pipeline like *SASFLOW*. After that, the results obtained from *p*(*r*) *versus r*, spatial modelling and other metadata (authors, beamline *etc.*) are incorporated. Finally, the sasCIF file can be submitted to a SAS database to be accessible for the community.

## 5. Conclusions   

This new version of the sasCIF format is intended to provide a major step towards standardizing the representation and exchange of SAS data and SAS-based models. The open source *sasCIFtools* developed for the processing of sasCIF files facilitate the conversion of the experimental SAS data files and models to the updated sasCIF format and *vice versa*. All major types of information used in SAS data analysis (scattering curves, distance distribution functions, models and fits of their calculated scattering to the experimental data) can be included in a single sasCIF file and easily exchanged. If needed, the two-dimensional area-detector data can also be included in a sasCIF file, using the imgCIF dictionary (Bernstein & Hammersley, 2005 ▸). The integration of the *sasCIFtools* into the SASBDB database (Valentini *et al.*, 2015 ▸) opens the possibility of online export and import of entire database entries encapsulated in a single sasCIF file. Following the wwPDB small-angle scattering Task Force recommendations (Trewhella *et al.*, 2013 ▸), these tools are a crucial element for data exchange between federated SAS databases. Implementation of sasCIF support in the *SASFLOW* pipeline (Franke *et al.*, 2012 ▸) allows beamline users to store their data in sasCIF format starting from the very first steps of data analysis. These measures, together with the introduction of SAS databases, make data organization and management more accessible for users, thus further promoting SAS applications in the structural biology community and improving the overall quality assurance and standards of the method. In this latest updated form, sasCIF can also be readily used for information exchange in non-biological SAS applications, and the format is easily extendable to accommodate the requirements of other communities (*e.g.* polymers or soft and hard condensed matter applications). 

## Supplementary Material

Supplementary tables and figures. DOI: 10.1107/S1600576715024942/aj5271sup1.pdf
