# Supplementary material for: Extension of the sasCIF format and its applications for data processing and deposition
Source: J Appl Crystallogr. 2016 Feb 1;49(Pt 1):302–10. doi: 10.1107/S1600576715024942 (PMC4762569; doi:10.1107/S1600576715024942)
Supplement: Supplementary file 1 [file j-49-00302-sup1.pdf]

JOURNAL OF APPLIED CRYSTALLOGRAPHY

Volume 49 (2016)

Supporting information for article:

Extension of the sasCIF format and its applications for data processing and deposition

Michael Kachala, John Westbrook and Dmitri Svergun

Table S1. Description of sasCIF category groups.

Group	Description	Members
beam_group	Categories that describe the properties of the beam.	sas_beam
detector_group	Categories that describe the properties of the detector.	sas_detc
fitting_group	Categories that describe the fitting of theoretical models' scattering to the experimental data.	sas_model_fitting<br>sas_model_fitting_details
intensity_group	Categories that describe the intensities	sas_axis<br>sas_scan<br>sas_scan_intensity
model_group	Categories that describe the models	sas_model
result_group	Categories that describe the results of the measurement	sas_result<br>sas_p_of_R<br>sas_p_of_R_details<br>sas_p_of_R_<br>extrapolated_intensity
sample_group	Categories that describe the properties of the sample.	sas_sample<br>sas_buffer

Table S2. Parent-child relations between for introduced categories

Parent	Child	Type of relationship
sas_sample	sas_buffer	One-to-one
sas_sample	entity	One-to-many
entity	struct_ref	One-to-one
sas_result	sas_scan	One-to-one
sas_result	sas_p_of_R_details	One-to-one
sas_p_of_R_details	sas_p_of_R	One-to-one
sas_p_of_R_details	sas_p_of_R_ extrapolated_intensity	One-to-one
sas_result	sas_model_fitting_details	One-to-many
sas_model_fitting_details	sas_model_fitting	One-to-one
sas_model_fitting_details	sas_model	One-to-many
sas_model	atom_site	One-to-one
citation	citation_author	One-to-many

Table S3. Correspondence between .dat file parameters and sasCIF data items

.dat file parameter	sasCIF data item
Scattering vector	`_sas_scan_intensity.momentum_transfer`
Scattering intensity	`_sas_scan_intensity.intensity`
Experimental error	`_sas_scan_intensity.intensity_su_counting`
Sample code	`_sas_sample.name`
Sample description	`_sas_sample.details`
Concentration	`_sas_sample.specimen_concentration`
Cell temperature	`_sas_scan.cell_temperature`
Storage temperature	`_sas_scan.storage_temperature`
Exposure time	`_sas_scan.exposure_time`
Timestamp	`_sas_scan.measurement_date`
Beam center position (x and y)	`_sas_detc.beam-position-x,.beam-position-y`
Wavelength	`_sas_beam.radiation_wavelength`

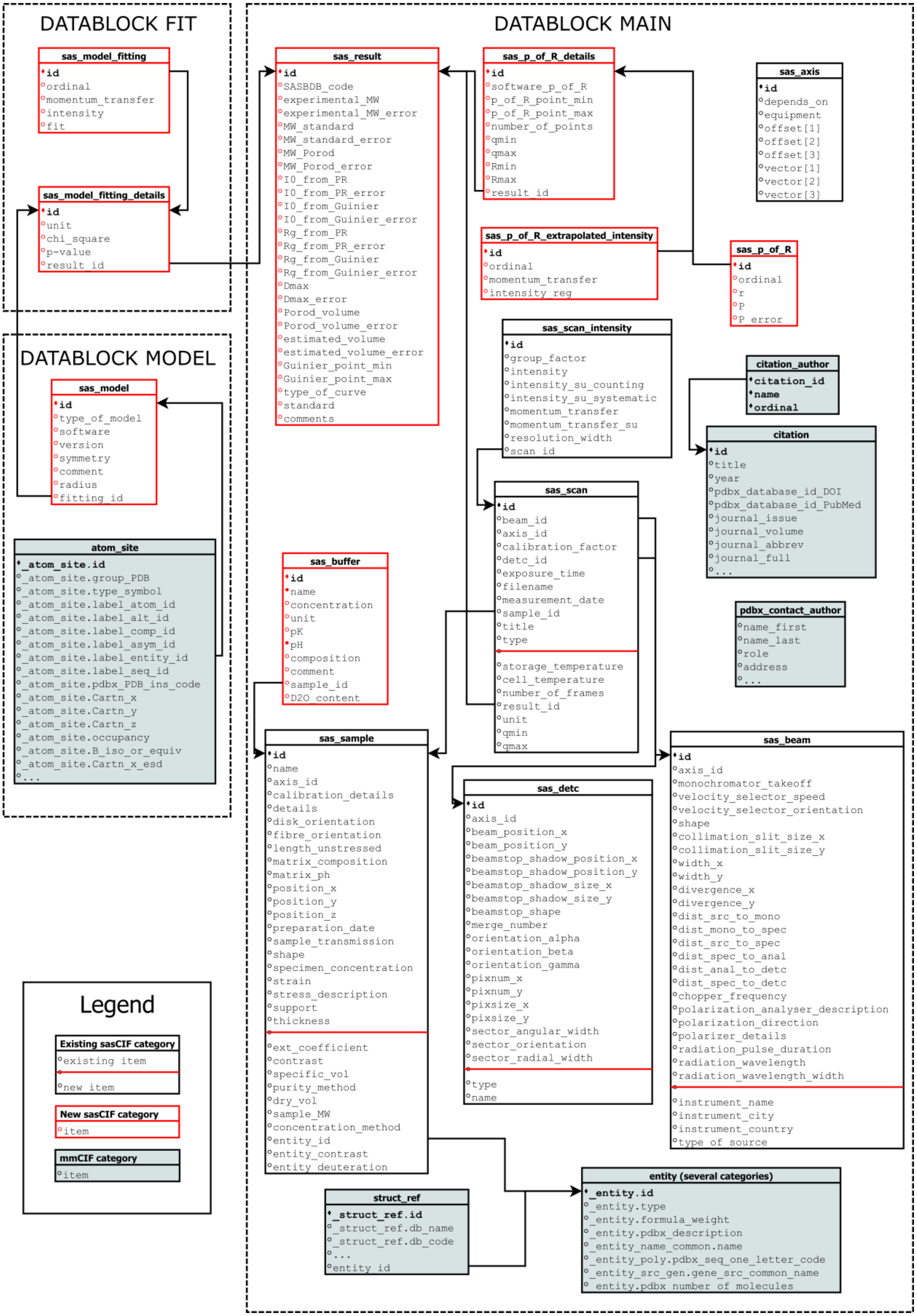

Fig. S1. A relational diagram of the updated sasCIF dictionary. The data items existing in the previous version(s) are shown in black boxes, while new categories are shown in red boxes together with their associated items. Items from mmCIF dictionary are in boxes with grey background.

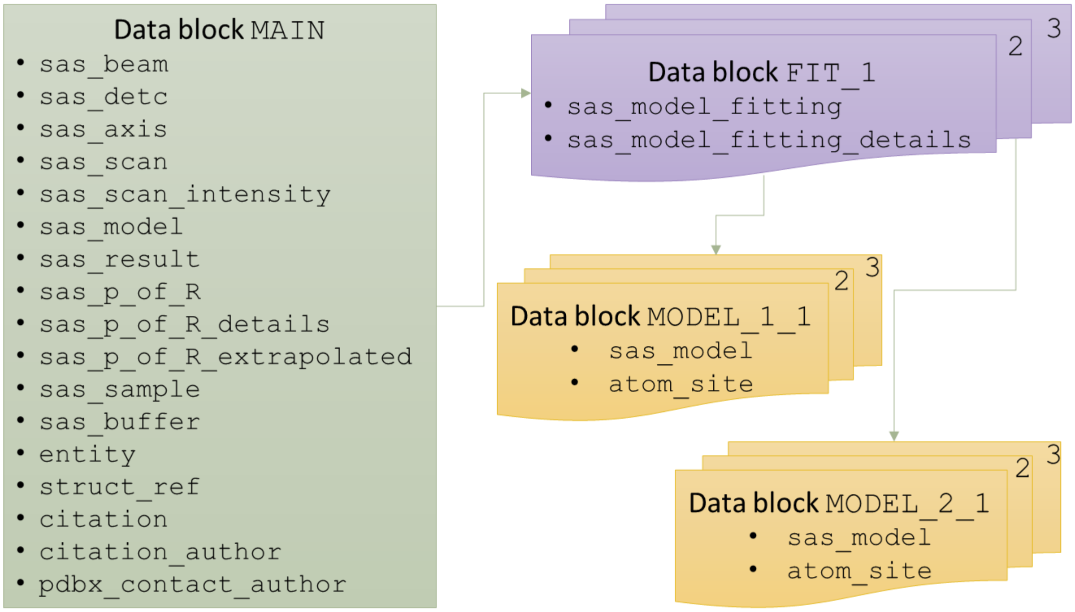

Fig. S2. Data block structure of sasCIF files.

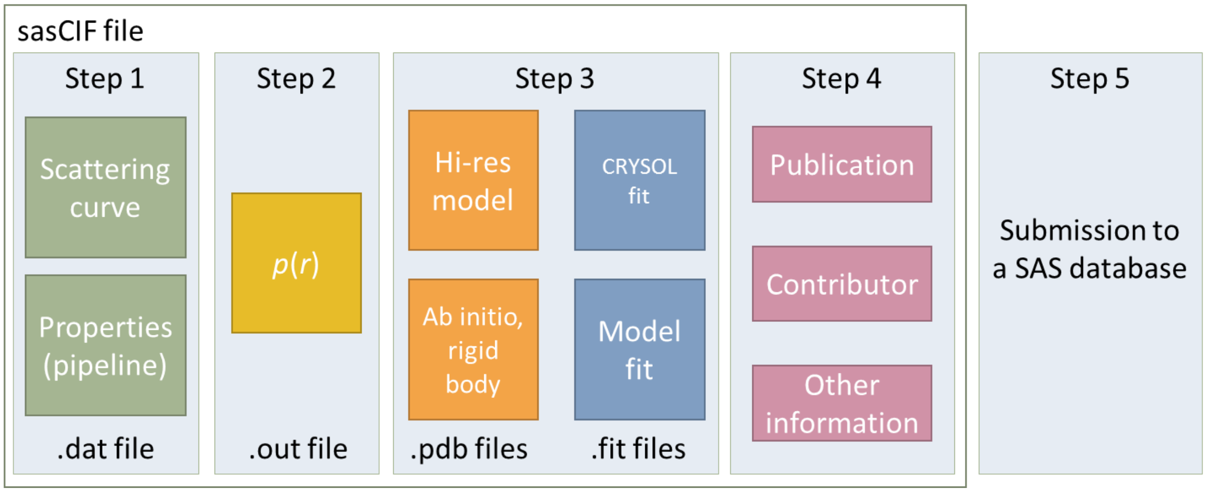

Fig. S3. Schematic use of sasCIF as a project file for SAS data analysis
